# Supplementary material for: Are carbon emissions trading and green financial instruments synergistic? -Comprehensive quantitative research based on content analysis
Source: PLoS One. 2024 Mar 7;19(3):e0298601. doi: 10.1371/journal.pone.0298601 (PMC10919601; doi:10.1371/journal.pone.0298601)
Supplement: S1 Data — (ZIP) [file pone.0298601.s001.zip › Data/地级市Z_F工作报告.zip!/╜¡╬≈/╔╧╚─╩╨/└┤╫╘╣1⁄2╓┌║┼í╛┬φ┐╦╩2╛▌═°í┐_2010.docx]

各位代表：

现在，我代表市人民政府，向大会报告工作，请予审议，并请市政协各位委员和列席会议的同志提出意见。

一、2009年工作回顾

过去的一年，是上饶在困境中化危为机，在挑战中克难奋进的一年。全市上下在省委、省政府和市委的正确领导下，深入学习实践科学发展观，认真贯彻胡锦涛总书记“三保一弘扬”指示精神，全面落实中央和省应对国际金融危机的一系列政策措施，牢牢抓住“保增长”这条主线，同心协力，积极应对，保持了经济社会平稳较快发展势头，较好地完成了市二届人大五次会议确定的目标任务。

（一）综合实力再上新台阶

全市生产总值达到 728.5亿元，增长13.6%；财政总收入达到83.5亿元，增长22.8%，其中一般预算收入48.1亿元，增长31.2％；完成固定资产投资612.4亿元，增长42.9%；社会消费品零售总额276.4亿元，增长19.4％；外贸进出口总额6.4亿美元，增长16%，其中出口5.8亿美元，增长23％；城镇居民人均可支配收入达到13989元，增长10.4%；农民人均纯收入达到4701元，增长8%。

农业经济全面发展。 稳定粮食生产，粮食播面830万亩，总产达到60.3亿斤，增长5.2％，连续六年创历史新高。积极发展多种经营，生猪、水产品、蔬菜、水果、油茶、茶叶等均保持了一定的增长。农民增收渠道继续拓宽，190家市级以上龙头企业实现销售收入147.9亿元，带动农户增收18.6亿元。信州区薄膜太阳能有机农业大棚一体化并网发电示范项目建成。2009江西·上饶农业产业招商暨农产品展示展销会成功举办。全面落实各项强农惠农政策，发放种粮农民“四补贴”6.5亿元。农业基础设施进一步完善。列入国家专项规划的147座病险水库除险加固工程全部开工，伦潭水利枢纽工程加快推进。完成中低产田改造10.3万亩，综合治理水土流失面积44.7万亩。新农村建设扎实推进，全年投入建设资金8.6亿元， 1334个试点村建设任务全面完成，151个乡镇、8316个村庄实施了垃圾无害化处理，新增户用沼气1.76万户。 农村公路建设完成2325.1公里， 行政村通畅率达到94.1%。

工业经济逆势上扬。 加大了工业园区建设力度，全市工业园区基础设施累计完成投资近20亿元，实际开发面积达到58.7平方公里，累计投产企业872家，园区企业实现主营业务收入770.2亿元，实现税金33.3亿元，从业人员13.8万人。上饶经济开发区和广丰工业园区主营业务收入分别达到185.4亿元和176.2亿元，位居全省十强园区第七、第八位。加大了企业帮扶力度，坚持把保企业作为保增长的关键，落实和出台了一系列政策措施，有力促进了企业健康发展，全市新增规模以上企业112户，年销售收入过亿元企业新增36户。千方百计加大工业投入，完成工业固定资产投资261亿元，增长44.5%。规模以上工业主营业务收入791.6亿元，增长21.7%，其中四大主导产业占71.7%；工业增加值达到292.8亿元，增长18.2%，其中规模以上工业完成增加值221.1亿元，增长20.4%；全市工业用电量41.5亿千瓦时，增长11.2%。

现代服务业繁荣活跃。 及时出台扩内需促消费政策措施，扎实推进旅游休闲、商贸物流基地建设，第三产业对经济增长的拉动作用明显增强。旅游业持续升温，灵山荣获国家级风景名胜区称号，新增3个国家4A级景区，龟峰申遗、三清山创5A级景区并申报世界地质公园等工作进展顺利。旅游景区服务功能不断完善。全年接待国内外游客1496.5万人次，增长49.5％。旅游总收入达到84.8亿元，增长39.5％。旅游总收入占生产总值的比重达到 11.6％。商贸物流业快速发展。步步高、洪客隆等一批知名商贸流通企业相继在中心城区落户开业。全市成交额过亿元的市场达到40家，新建和改造农家店1100家。家电下乡产品销售额、补贴额均位居全省前列。金融服务创出佳绩。全市金融机构年末存款余额781.8亿元，增长28%；贷款余额499.8亿元，增长36.9%，新增贷款134.8亿元。上饶市商业银行更名为上饶银行，并在鹰潭建立分行，迈出了从地方银行向区域银行转变的重要一步。引进外资银行、股份制银行工作取得突破，民生银行在上饶设立分支机构进展顺利。同时，房地产、保险、邮电通讯、烟草、石油等产业健康发展。

县域经济势头良好。 各县（市、区）加快推进工业化、城镇化和农业产业化，形成了各具特色的产业布局，呈现出比学赶超、加快发展的喜人局面。12个县（市、区）财政均超过3亿元，其中广丰、德兴过13亿元。财政收入超亿元的乡镇达到9个，较上年增加5个。市本级（含经济开发区、三清山风景名胜区）财政收入达到10.6亿元。

（二）项目建设实现新突破

全力组织“双千”工程大会战,主攻项目，决战项目。全年共调度续建和新建项目822个，竣工153个，完成投资368亿元。

重大基础设施项目快速推进。 峰福铁路电气化改造顺利竣工，合福客专、杭南长客专已经开工，皖赣铁路改造、九景衢铁路前期工作正在抓紧进行；上武、德昌高速公路快速推进，德上高速公路即将开工建设；三清山机场各项前期工作进展顺利，有望上半年开工建设；境内320国道路面重建工程全面完成；64座渡改桥工程全部开工，11座建成通车；天然气入饶项目已经动工，黄金埠电厂二期正在全力争取国家发改委核发“路条”，上饶电厂项目初可报告已通过专家评审；改建和新建11万伏以上变电站11座，鄱阳和铅山22万伏输变电工程进展顺利。“数字上饶·无线城市”战略快速推进，移动电话用户突破260万户，互联网用户突破20万户，固定电话用户突破100万户。

重大产业项目强势推进。 全市新增投资5000万元以上工业竣工项目99个。晶科能源200兆瓦太阳能电池组件建成投产。鄱阳凯迪能源2×12兆瓦机组并网发电，和丰铜业主营业务收入突破30亿元，金汇铜业一期、大自然木业一期、锦华电脑刺绣机、华晟金属、东海食品、博盈能源、聚芳永名优茶等一批重大产业项目建成投产。

社会事业项目协调推进。 争取到国家扩内需社会事业类项目投资3亿元，投入教育、卫生、文化、计划生育等社会事业。市图书馆、科技馆、特教学校、红十字会备灾仓库、麻风病康复中心全面竣工，上饶医学分院新校区主体工程、市福利中心、社会救助站基本完工。弋阳 方志敏纪念馆、怀玉山北上抗日先遣队纪念碑、上饶集中营旧址整修、信江书院改造基本完成。

（三）城镇面貌发生新变化

城镇规划科学合理。 中心城区总规修编、上饶市土地利用总体规划修编通过省政府审批，商业发展规划业已颁布实施，旅游发展总规修编通过专家评审。中心城区建设用地范围内的控规和专项规划进一步完善。弋阳、铅山等地总规修编基本完成。一批重点镇的规划正在抓紧编制。

城镇建设如火如荼。 加大了中心城区重点城建项目推进力度，16项民生工程基本完工，28个重点工程已全面开工，累计完成投资35.6亿元。广信南、北大道、凤凰东大道、庆丰路、志敏大道一期、叶挺大道南北段、明叔路、双塔公园至樱花公园连接段等8条中心城市主次干道建成通车，丰溪大桥、胜利大桥改造、奥林匹克中心、艺术中心以及20万吨自来水厂、龙潭湖综合整治等重点项目加快推进。步行街人行天桥、老城区小街小巷改造、污水管网、下水道清改，云碧峰森林公园游步道等一批方便市民生活的项目全面完工。中心城区建成区面积达到51.27平方公里，规划区人口达到47.8万人。与此同时，县城建设呈现出大框架、大项目、大生态的发展态势，全国重点镇达9个，省重点镇达27个，国家历史文化名镇（村）2个。全市城镇化率达到35.5%，比上年提高了两个百分点。

城市管理成效明显。 积极推进城市管理科学化、规范化和法制化，进一步加大了投入、理顺了体制、完善了体系，城市管理的长效机制逐步建立。深入开展了中心城区“十乱”和“两违”整治活动，积极推动“市民看城市、评创建”和“告别陋习，树立新风”主题教育活动，广大市民关注城市管理、参与城市管理的热情空前高涨，省文明城市创建取得积极成果，市容市貌进一步改观。各县（市、区）加大创建工作力度，城镇管理水平不断提高。信州区荣获“全国和谐社区建设示范城区”称号。

（四）改革开放增添新活力

各项改革不断深化。 全力 加大国有企业改革攻坚力度 ，全市32户工业企业改制基本到位；医药卫生体制改革有序推进，基本药物制度试点工作正式启动；政府机构改革稳妥推进，其它各项改革不断深入。

开放型经济成果丰硕。 把招商引资作为项目建设最关键的环节，精心组织，高位推动， 产业招商、会展招商、小分队招商、以商招商成效显著 。积极参加了合肥中博会、香港活动周、赣台经贸会、世界低碳（南昌）大会等活动，取得丰硕成果。 与山东淄博、烟台，浙江宁波，福建南平、福州、宁德等城市，建立了较为紧密的协作关系。全年实际利用外资3.5亿美元，增长16%；新引进省外5000万元以上工业项目资金132.8亿元，增长26.3%。其中新引进亿元以上工业项目38个，实际进资66.3亿元，进资总量创历史新高 。 上饶“无水港”正式运营。资本运作实现突破，万年江晨控股在香港创业板成功上市。

全民创业方兴未艾。 以推进“双万”工程为载体， 深入开展了“双创”活动，着力推进创业富民。全市 农家乐经营户达到12702家； 净增个体工商户14851户，新增私营企业2550户，注册资本达到47.5亿元。

（五）保障能力有了新提高

把保障和改善民生作为保增长的目的、促和谐的重点，加快推进民生工程实施步伐。全市90项民生工程指标达到任务要求，涉及群众切身利益的 60件实事顺利完成。就业再就业不断扩大。全市城镇新增就业人数10.3万人，新增转移农村劳动力15.3万人。6.5万名农村劳动力接受了培训，零就业家庭就业安置率达到100%，11823名大学毕业生实现了就业，390名残疾人得到了公益性岗位，30万人参加了失业保险，为就业创业提供小额贷款4.7亿元。社会保障体系进一步完善。城镇居民医保基本实现了全覆盖，在校大学生全部参保，城镇职工基本医疗保险参保人数达到61.5万人，国有关闭、破产、改制及困难企业退休人员由财政补助参保。新农合参保农民达到514.4万人，一次报账率达到94.6%；城镇职工参加基本养老保险60万人，7.3万名被征地农民参加了养老保险，婺源县被列为全国首批新型农村养老保险工作试点县；38万名城乡困难群众得到了最低生活保障，发放低保金达4.6亿元；新增“三院”建设面积6.4万平方米，农村“五保户”集中供养率进一步提高。困难群众老有所养、病有所医的愿望逐步成为现实。保障性住房建设扎实推进。全市经济适用房开工建设12.1万平方米，已竣工6.9万平方米；开工建设廉租住房5873套，已竣工2140套。有16771户得到了租赁补贴，一批低收入群众圆了住房梦。改造棚户区74.9万平方米，已建成面积36.1万平方米。为农村困难群众新建、重建住房1126户。就医助学力度加大。共发放城乡困难群众医疗救助资金1.1亿元，免费提供社区公共卫生服务卡368万元；免除了104.4万名学生的学杂费并免费提供教科书，8万名义务教育阶段贫困寄宿学生、1.2万名普通高中贫困学生、2.2万名中等职业教育学生得到了资助， 扶助2816名寒门学子圆了大学梦。扶贫开发加快推进。全年共投入扶贫专项资金1.2亿元，完成整村推进项目409个，有6064名移民喜迁新居。

（六）社会事业取得新进步

各项社会事业协调发展。 义务教育得到加强，职业教育健康有序，民办教育不断规范，中小学校舍安全工程全面推进。科技创新“633611”工程稳步实施，科技入园取得实效。文体活动好戏连台，第二届市运会成功举办，央视心连心艺术团演出圆满成功，信州区荣获全国先进文化社区称号，玉山县荣获“中国民间文化艺术之乡”称号。卫生事业加快发展，市人民医院创“三甲”医院获得成功，甲型H1N1流感等传染病防控工作取得阶段性胜利。“光明·微笑”工程有效推进， 17760例白内障患者重见光明，858名唇腭裂患者露出微笑。食品药品专项整治扎实开展，全市未发生重大食品药品安全事件。人口计生工作水平不断提高，奖励优惠政策全面落实，人口自然增长率控制在7.97‰以内，出生率下降1.25‰，出生人口性别比日趋合理。民族宗教工作取得新成绩，铅山县被国务院授予全国民族团结进步模范集体荣誉称号。深入开展国防教育，部队和民兵预备役建设得到加强，军政军民关系进一步巩固。新闻出版、统计、审计、地方志、档案、保密、外事侨务、涉台事务、防震减灾、人民防空、社会福利、妇女儿童、慈善、红十字、老龄、残疾人工作等各项事业取得了新成绩。

生态建设扎实推进。 围绕鄱阳湖生态经济区建设，大力推进造林绿化 “ 一大四小 ” 工程，共投入建设资金15.6亿元，植树造林90万亩，通道绿化近9000公里，被评为全省先进设区市。出台了《上饶市信江城区段饮用水源保护管理实施意见》、《信江上游产业发展布局的环境保护意见》，饮用水安全保障长效机制逐步建立。深入开展节能减排工作，严格环境评估，强化源头控制，果断否决了16个不符合国家产业政策、对环境造成污染的项目。加快环保设施建设，十个县（市）污水处理厂全部竣工。主要污染物二氧化硫和化学需氧量分别减排3466吨和1494吨，提前一年完成“十一五”静态减排任务。

（七）安全稳定呈现新局面

安全生产形势平稳。 深入开展“安全生产年”活动，注重宣传教育，狠抓源头治理，严格责任落实，各类事故起数下降29%，死亡人数下降13.7%。

信访工作力度加大。 广泛开展普法教育和法律服务，认真开展“信访积案化解年”活动，加快构建信访信息综合服务系统，高度重视人民来访接待中心建设，进一步规范领导定期接访和包案处理制度，有效化解了一批重点疑难信访问题，信访态势总体良好。

社会治安不断加强。 深入开展社会治安综合治理专项行动，完成中心城区“天网工程”建设，防控体系不断健全，群防群治局面逐步形成，治安乱点、盲点得到了有效整治。全力做好了建国60周年庆典活动和其它节庆活动期间的安保工作，公众安全感进一步提高，社会政治大局稳定和谐。

（八）自身建设展示新形象

坚持依法行政，按照法定权限履行职责，自觉接受人大法律监督、政协民主监督以及新闻媒体等社会监督。坚持科学决策、民主决策，广泛听取各民主党派、工商联、无党派人士以及各人民团体的意见和建议，充分发挥社团、行业协会和中介组织的积极作用。认真办理人大代表建议和政协提案，满意率达96.7%。深入开展学习实践科学发展观活动，针对群众提出的涉及政府工作的七大类共38个问题，全部制定了整改方案，并限时解决。扎实开展机关效能年活动，下大力气减少行政审批，提高办事效率，精简市本级行政许可项目131项、投资项目审批事项91项。牢固树立过紧日子思想，公务用车、会议经费、公务接待费用及党政机关出国（境）经费实现了“零增长”。严格落实廉政责任制，突出了重点领域廉政制度建设，行政监督体系进一步健全。

各位代表，刚刚过去的2009年，我们共同庆祝了新中国60华诞，共同经受了国际金融危机的严峻挑战，共同分享了改革发展的喜人成就。这些成绩的取得，是省委、省政府和市委正确领导的结果，是人大政协、老干部、驻市单位和社会各界监督支持、理解帮助的结果，凝结了全市人民的智慧、汗水和力量。在此，我代表市人民政府，向全市广大工人、农民、知识分子、干部和离退休老同志，向各民主党派、工商联、无党派和社会各界人士，向驻饶部队、武警官兵和公安干警，向广大海内外投资者和所有关心支持上饶经济建设、社会发展的同志们、朋友们，表示衷心感谢并致以崇高敬意！

回顾一年来的工作，我们也清醒地认识到，全市经济社会发展还存在着一些困难和问题。主要是：经济总量偏小、人均量偏低、财政实力较弱的现状尚未根本改变；三农工作基础仍不牢固，农民持续增收的难度依然较大；支撑经济发展的大项目、大企业不多，自主创新能力亟待提升；社会保障体系尚不完善，保障和改善民生的任务依然繁重；政府管理还存在一些薄弱环节，影响社会和谐稳定的因素依然存在；公务员队伍服务意识和办事效率有待进一步提高，政府工作与人民的期望还有一定差距。对这些问题，我们一定会高度重视，认真对待，在今后的工作中逐步加以解决。

二、2010年工作目标

2010 年，是实施“十一五”规划的最后一年，也是我市撤地设市十周年，更是我市实现新发展、再上新水平的关键一年。在这样一个重要的年份，我们必须进一步增强忧患意识、机遇意识和责任意识，进一步弘扬方志敏精神，始终保持进取精神和拼搏勇气，一刻也不懈怠，一天也不耽误。今年政府工作的总体要求是： 全面贯彻党的十七大、十七届三中、四中全会、中央和全省经济工作会议精神，以邓小平理论和“三个代表”重要思想为指导，深入贯彻落实科学发展观，紧紧围绕“打造一个枢纽、建设四个基地”发展格局，致力于促进“三个转变”，着力推进“两个跨越”，切实提供“一个保障”，在调结构、促转变、抓改革、增后劲、惠民生上下功夫，努力推动上饶科学发展、和谐发展、跨越发展。

今年全市经济社会发展主要预期目标建议为： 生产总值达到835亿元，增长12%；财政总收入96亿元，增长15%;固定资产投资810亿元，增长32%；社会消费品零售总额330亿元，增长19.4%;城镇居民人均可支配收入增长10%,农民人均纯收入增长8.5%;人口自然增长率控制在8‰以内;城镇登记失业率控制在4.5%以内； 城镇化率提高2个百分点；每万元生产总值能耗、电耗、化学需氧量、二氧化硫排放量严格控制在省政府下达的指标之内。全面完成“十一五”规划的各项目标任务。

上述目标，是贯彻落实中央、省委和市委大政方针的基本要求，是保持良好势头、谋求新一轮发展的现实需要，是我们实现跨越发展、争先进位的必然选择。完成上述目标，我们面临着许多有利条件和难得机遇。当前全球经济已经出现恢复性增长迹象，我国经济回升向好的态势比较明显，国家宏观经济政策保持连续性和稳定性、提高针对性和灵活性，为我们加快发展提供了良好的外部环境。 鄱阳湖生态经济区建设上升为国家战略，这是江西发展史上的重要里程碑，更是我们实现科学发展、和谐发展和跨越发展千载难逢的重大历史机遇。加之上饶既是海西经济区重要成员城市，又是长三角经济区的腹地， 三大经济区各种要素聚合和“叠加效应”，必将为上饶的发展注入强大动力。更为 重要的是， 历届班子打下了良好的基础，在新的历史起点上，我们的发展思路更加清晰，发展目标更加明确，发展 举措更加实在，全市广大干部群众奋勇争先、进位赶超的信心更加坚定 。 特别是在应对国际金融危机冲击的重大考验中，我们积累了在复杂环境下推动经济社会又好又快发展的宝贵经验，我们完全有信心、有条件、有能力实现上述目标。

三、2010年工作重点

围绕上述目标，我们要重点抓好十个方面的工作：

（一）以项目建设为引领，力促经济发展增实力、增后劲

项目建设是经济工作最核心的内容和最重要的抓手，是保增长、强实力、添后劲最重要的载体。我们要加快推进“双千工程”，千方百计上一批大项目、好项目。

继续抓好重大基础设施建设。 积极配合抓好合福客专、杭南长客专、九景衢铁路和皖赣铁路建设工作，加快上武、德昌高速公路建设，开工建设上饶三清山机场、德上高速公路；全力争取黄金埠电厂二期项目，抓紧上饶电厂项目前期工作；加快与海西经济区的对接，积极筹建宁德港上饶码头项目，建设出海通道；抓好天然气入饶项目实施；继续抓好通讯、电力等基础设施建设。加快3G网络 “数字上饶·无线城市” 建设。进一步加强农业基础设施建设，全面完成中型和小（一）型水库除险加固工程，加快实施伦潭水利枢纽，继续实施七一、饶丰、鄱湖三大灌区续建配套和节水改造工程，加快实施1-5万亩圩堤加固工程。

加快推进重大产业项目建设。 继续抓好晶科能源第3条100兆瓦太阳能电池组件、德兴铜矿日采选13万吨矿石扩建、弋阳海螺第2条日产4500吨水泥生产线、银山矿业日采选5000吨矿石技改工程、索普信年产5000万支高效环保稀土节能灯、和丰铜业年产18万吨阳极铜等一批续建项目。加快推进昂大集团年产1000万只铝合金汽车轮毂生产线、新陆地年产240兆瓦电池片和400兆瓦组件生产线、金山金矿日采选6500吨矿石扩建、圣达威电工年产6万吨铜杆及铜铝漆包线、江钨集团年产2万吨钴镍新材料、雄安金属年产10万吨铜合金棒和硅铝合金、中山雄兵年产2万吨再生橡胶制品、佳华二期年产50万台电冰箱（柜）、天佑科技年产8000吨精制茶油、海力电器年产20万台洗衣机以及华邦药业、韦伯尔铝业等一批新建项目。保持工业投资较快增长，关键是依靠招商引资和激活民间资本。为此，要强力推进招商引资，围绕四大主导产业，加快洽谈一批、签约一批、开工建设一批固投10亿元以上或达产达标后主营业务收入10亿元以上的产业项目。力争工业固定资产投资占全部固定资产投资的比重达到50%。

协调推进各类社会事业项目。 继续抓好奥林匹克中心、创意产业中心、上饶医学分院新校区 建设。 开工建设上饶中学、上饶市生态博物馆和 市精神卫生中心 ，启动上饶市城市防洪沽塘蓄涝区（碧玉湖公园）建设，加快新一代雷达信息处理中心建设。

（二）以鄱阳湖生态经济区建设为主线，力促经济社会发展更好、更快

建设鄱阳湖生态经济区，是惠及当代、泽被子孙的大工程，是引领长远发展的大战略，是加快崛起的大机遇。鄱阳湖生态经济区建设，特色是生态，核心是发展，关键是转变发展方式，目的是走出一条科学发展、绿色崛起之路。作为鄱阳湖生态经济区规划内的重要一员， 我们一定要深刻领会鄱阳湖生态经济区建设的本质内涵，坚定不移地把建设鄱阳湖生态经济区作为推动上饶“走绿色崛起之路、建生态和谐之城”的重大战略和龙头工程，积极策应，精心组织，加快推进，全面融入，努力争当鄱阳湖生态经济区建设的先行区和排头兵。

做好规划对接和实施。 加强与省直有关厅局专项规划的衔接，积极争取省直厅局在规划、政策、项目和资金等方面的指导和支持。加快推进鄱阳湖生态经济区重大项目的组织实施，重点在新能源、新材料等方面尽快启动一批重大项目，引领和带动全市新兴产业的跨越发展。

加快构筑生态产业体系。 大力发展低碳经济，突出技术含量高、资源能源消耗低、污染物排放少、人力资源优势得到充分发挥的产业，重点推进新能源、新材料产业以及循环经济向纵深发展，加快构筑生态工业体系。围绕打造优质农产品加工供应基地目标，大力推进无公害、绿色、有机农业发展，构建高产、优质、高效、安全的生态农业体系。充分发挥生态资源和交通区位优势，加快发展以生态旅游、特色文化、商贸物流为重点的现代服务业。

加大节能减排力度。 加快实施建筑节能、绿色照明等重点节能工程，推进重点领域和能源、原材料等重点行业节能，抓好公共机构节能。加大火电、有色金属、水泥等重点行业污染物减排力度，严格执行达标排放。继续推进县市污水处理设施配套建设，重点加强污水管网配套，提高运营效率。加快工业园区污水处理设施建设，集中处理园区内工业和生活污水。加大信江、乐安河流域和鄱阳湖水体保护，进一步强化环境监测，保障城乡居民饮用水安全。确保全市主要污染物二氧化硫和化学需氧量分别减排2500吨和1560吨；万元工业增加值能耗下降5.5%，工业固体废物综合利用率达到4.3%。

大力推进绿色生态建设。 围绕通道绿化、城区绿化和农村绿化三个重点，深入推进百万果苗进新村、打造千里精品走廊、建设万亩城市森林三大工程，加快推进造林绿化“一大四小”工程，确保完成造林50万亩以上。加大水土流失治理力度，完成治理水土流失40万亩。加大国家森林公园创建力度，加快推进鄱阳湖湿地公园建设。 加速推进中心城市绿化和生态建设步伐，同步创建 国家园林城市和国家森林城市。

大力提升科技创新能力。 以实施科技创新“633611”工程为重点，进一步加大科技投入， 完善 科技服务体系， 支持技术含量高、市场前景好的科技型企业开展自主创新。大力推进科技创新平台建设，加强产业研发中心、技术服务中心、产品检验检测中心、人才培训中心等公共服务机构建设，支持企业依靠科技进步和科技创新，提高劳动生产率、人均创利率、资金利润率、资源能源利用率。加大高科技人才引进力度，积极开展与国内外权威科研机构、大学的深度合作。大力开展科技进村入园工作，积极推广先进适用技术，不断提升科技对经济增长的贡献率。

（三）以产业发展为支撑，力促新型工业化上速度、上效益

围绕做大总量、调优结构、提升效益的目标，全力加快推进新型工业化。力争实现全部工业增加值350亿元，增长18%，其中规模以上工业增加值达到265亿元，增长20%。

增强园区承载能力。 坚持规划先行、 基础设施先行，着力形成一批“功能完善、管理规范、环境优美、特色鲜明”的工业园区。强化园区软环境建设，加强对“一区十园”的调度和服务，加快推进特色产业基地建设。实现上饶经济开发区、广丰工业园主营业务收入突破200亿元，力争横峰和玉山工业园主营业务收入突破100亿元，其它工业园区力争进入全省二类园区。力争全市工业园区主营业务收入突破1000亿元、利税突破100亿元、入园企业突破1000户，招商引资到位资金达到200亿元，园区从业人员达到18万人。

促进产业升级。 着力提升以铜材精深加工、黄金采选加工和铅锌冶炼为主的有色金属产业，以光学、汽车为主的机电光学产业，以干法水泥为主的新型建材产业的综合竞争能力，促进传统产业转型升级。加快培育以光伏产业为主的新能源产业，使其成为工业快速发展的增长极。积极推动产业集群发展，围绕四大主导产业，着力扶持20家产业关联度高、核心竞争力强、带动效应明显的龙头企业，延伸产业链，扩大集聚规模，提升竞争实力。

扶持优强企业。 继续完善支持工业企业发展的政策措施，不折不扣地抓好各项政策的贯彻落实。继续抓好销售收入过10亿元的重点企业和投资过5亿元的重点项目的调度分析。加快已改制企业与央企、省企和民企的对接，积极引进战略投资者，促进已改制企业焕发生机，做大做强。力争全市主营业务收入达50亿元的工业企业实现零的突破，过30亿元的企业达到2户，过10亿元的企业达到8户，过亿元的企业达到190户，规模以上工业企业突破800户。

（四）以新农村建设为载体，力促“三农”工作基础更扎实、更牢固

围绕稳粮保供给、增收惠民生、改革促统筹、强基增后劲，加快推进国家粮仓和优质农产品加工供应基地建设，确保粮食生产不滑坡、农民增收不徘徊、农村发展好势头不逆转。

稳定发展粮食生产。 扎实推进高产示范和良种良法推广等项目建设，积极引导土地承包经营权有序流转，进一步强化耕地和基本农田保护，稳定面积、优化品质、提高单产，确保全年粮食总产稳定在60亿斤以上。全面落实强农惠农政策，突出做好防汛抗旱、畜禽防疫、森林防火、农资防假工作，夯实农业安全基础，提升农业安全水平。

加速推进现代农业。 加快高效生态农业建设，大力推进水稻、生猪、水产、茶叶、水果、油茶、毛竹等优势产业区域化、标准化、规模化发展，建立20个标准化优质农产品生产基地。大力引进、培育和扶持农业龙头企业，新增规模以上农业企业30家，销售收入10亿元以上龙头企业2家。不断提高农业生产的组织化程度，新增注册农民专业合作社500家以上。继续办好农业产业招商和农产品展销活动，尽快启动上饶（台湾）现代农业产业园项目，努力打造吸引台湾资本、对接台湾农业、融入海西发展的一体化农业发展平台，不断提高农业开放水平。

着力推进新农村建设。 抓好1128个新农村试点村建设，突出规划引领、产业发展、绿化美化和特色提升，加大资金筹集和环境整治力度，加快建设一批湖区生态渔村、景区旅游新村、城郊社区新村，继续抓好试点村主干道硬化、改水、改厕等实事。按照“减量化、资源化、无害化”的要求，加快农村垃圾处理基础设施建设，扎实推进农村清洁工程，积极推进行政村邮站建设。

（五）以完善功能为重点，力促新型城镇化出亮点、出精品

深入实施“双轮驱动”战略，坚持高起点规划、高标准设计、高水平经营、高强度建设、高效能管理，加快推进新型城镇化步伐，把上饶打造成宜居宜业宜人的绿色生态城市。

坚持规划引领。 严格执行新修编的《上饶市城市总体规划》，科学编制中心区控制性详规和城市地下管网、城市交通、城市亮化、绿地系统、水系规划以及湿地开发保护等专项规划。加快县城总体规划、控制性详规编制工作，积极推动 重点镇规划编制工作。

坚持项目带动。 按照“高起点、高标准、高质量、高品位、高速度和规范化、人性化、个性化、数字化、生态化”的要求，加快龟峰大道、天佑大道、志敏大道 二期、龟峰大桥等主干道建设，进一步拓展城区框架。加快完善中心区、带湖片区、三江口片区路网建设工程，推进辛弃疾公园、五桂湖公园、龙潭湖公园、槠溪湿地公园建设。加快行政中心南北绿色中轴线、凤凰大道、广信大道等精品景观线路建设，不断提升城市品位。继续抓好居民小区、小街小巷的硬化、亮化、美化和地下管网、地上基础设施的配套完善工作，努力让广大市民出行更方便、居住更舒适、心情更舒畅、生活更美好。

坚持机制创新。 围绕创建国家园林城市和文明城市，深入开展“城市管理攻坚年”活动，不断完善城市管理长效机制。深入开展“两违”整治工作，做到发现一起、处治一起，将违法建筑、违规用地现象遏制在源头上，处理在苗头上。高度重视社区工作，进一步完善社区管理和服务体系。认真组织好各项创建活动，深入开展“告别陋习，树立新风”活动，组织和动员广大市民参与到城市管理中来，努力形成“人民城市人民爱、人民城市人民管”的浓厚氛围。

坚持整体推进。 按照“一级中心、二级重点、三级传递、四级网络”的发展要求，构建“中心城市—县城—重点镇—中心村”为骨架的城镇化梯度发展格局，着力加快“1+5”信江河谷城市群建设，形成科学合理的城镇体系，促进城乡协调发展。加快县城和示范镇建设，确定30个市级示范镇，重点扶持，加快推进。进一步 扫除制度性障碍，降低城镇门槛，吸引人口进城，增强城镇的人口集聚能力，确保全市 城镇化率达到37.5% 。

（六）以扩大消费为途径，力促商贸旅游业更活跃、更繁荣

扩内需、调结构仍然是今年经济工作的重点。 围绕建设全国旅游强市目标， 加快转变旅游产业发展方式，全面提升我市旅游产业发展水平，加快发展商贸物流业，不断 繁荣第三产业，提升三产对经济增长的贡献率。

加快旅游休闲基地建设。 实施旅游品牌创建战略，继续做好龟峰申报世界自然遗产、三清山申报世界地质公园工作，加快推进三清山、婺源、龟峰创5A级景区步伐。大力推动玉山、万年、弋阳、铅山创建全省旅游强县，加快启动三清山、婺源创建国家级旅游标准化示范点等工作。完善旅游服务设施，提升中心城区的旅游接待、服务能力，把中心城区建设成为旅游交通节点城市和游客集散中心。 加快推进三清山枫林服务区建设，完善婺源、玉山旅游服务区功能，使之与中心城区的集散能力互为配套，互为依托，互为补充。加强市内外旅游线路的整体策划和包装，做好旅游行业的沟通对接，推进旅游区域合作、互联互通，实现无障碍旅游。 大力发展农家乐，加强服务指导，规范经营行为，使之成为农民增收的一条重要渠道，力争新增农家乐经营户5000家。全年实现旅游总收入105亿元，增长28%；接待国内外游客1800万人次，增长20%。

增强消费对经济增长的拉动力。 加大商贸物流业招商引资力度，积极引进更多知名企业入驻上饶，使中心城区商贸服务业火起来、旺起来。规范有序发展房地产业，有效调控房地产市场，合理引导商品房开发。积极推进连锁便利店、经济型住宿、餐饮等社区服务业发展，着力扩大社区消费。继续推进“万村千乡市场工程”，扎实做好家电、汽车、摩托车下乡工作，努力扩大农村消费。

（七）以增动力、添活力为目的，力促改革开放更深入、更广泛

没有改革就没有动力，没有开放就没有活力。只有从更深层次深化改革，从更宽领域扩大开放，才能不断创新机制，增强抵御风险的能力，实现上饶的长远发展。

继续深化重点领域改革。 有序推进农垦、粮食、农业、水利、林业、商贸流通、交通运输等系统 国有企业改革， 全面完成“三个到位”目标。 加快推进医药卫生体制改革， 巩固和完善城镇职工基本医疗保险、城镇居民基本医疗保险和新型农村合作医疗制度。加快 建立国家基本药物制度，认真抓好试点工作，切实减轻广大群众基本用药费用负担。 全面完成市、县政府机构改革。 稳步推进供销社改革。 启动文化体制改革，加强公共文化服务体系建设。

不断提升对外开放水平。 充分发挥口岸平台功能， 办好上饶“无水港”，完善综合保税功能，推进铁海联运常态化。继续争取上饶经济开发区升格为国家级经济技术开发区并设立出口加工区。 继续 加大产业招商力度，围绕重点产业，瞄准重点地区，继续加强与世界500强、国内500强、台湾100大企业的联系和对接， 确保全年实际利用外资4亿美元，增长15%，现汇率达到50％；引进省外单项投资5000万元以上工业项目实际进资159亿元，增长20%。用活用好国家、省外贸出口发展基金，大力发展符合国家产业政策导向的加工贸易主体，支持骨干企业扩大出口规模，增强出口企业发展活力。加强与国内、国外友好城市对接，进一步扩大经贸、文化、旅游等方面的交流与合作。全年实现外贸进出口7亿美元，增长10%，其中出口6.4亿美元，增长10%。

积极创新投融资体制。 创新银企合作平台，深化政银企融资对接。引进国内外股份制银行来饶设立分支机构。做大做强上饶银行、农村信用社等地方银行。稳健发展村镇银行、小额贷款公司。加强金融产品创新，加大对产业发展、全民创业、个人消费等领域的金融支持力度。建立多层次、多元化的中小企业信用担保体系，担保总额达到5亿元以上。大力发展资本市场，推进企业股份制改革和上市，力争年内有1-2户企业在境内外上市，实现融资能力的新提升。

大力促进民营经济发展。 深入开展“双创”活动，进一步放宽市场准入，扩大民间投资领域，鼓励和支持外出务工经商人员返乡创业。 积极引导中小企业健康发展，鼓励和支持中小企业与大企业建立稳定的原材料、零部件供应等方面的协作关系。 支持中小企业进入生产性服务、装备制造业和高新技术产业，积极发展中小企业集群，提高中小企业集约化发展水平。力争全年新增个体工商户1.7万户以上、私营企业2400家左右、私营企业注册资本达到40亿元以上。

（八）以改善民生为根本，力促保障体系更健全、更完善

民生连着民心，民心凝聚民力。保障和改善民生，是各级政府的重大责任，把事关群众切身利益的好事办好，实事办实，把群众满意、群众高兴作为各级政府的最大追求。

一是积极扩大就业。 坚持更加积极的就业政策，加大力度做好农民工、大学生和零就业家庭的就业工作。 继续推进“阳光工程”、“雨露计划”、“金蓝领工程”， 实现城镇新增就业4.7万人，城镇净增就业4.3万人，下岗失业人员再就业2.2万人，“4050”人员再就业2800人，城镇就业率94.5%，继续保持零就业家庭动态清零；新增转移农村劳动力7.9万人，其中省内转移4.5万人。让就业是民生之本方针在更多的政策措施上得到落实。

二是健全社会保障体系。 继续扩大各类社会保险覆盖范围，重点推进农民工、非公有制经济组织就业人员、个体工商户、灵活就业人员参保；调整企业职工基本养老金待遇水平，并确保100%足额发放；做好解除劳动关系职工的养老保险接续工作，力争新增参保职工5万人，实际缴费职工人数占参保职工总人数的90%以上；继续巩固工伤保险、女工生育保险工作，力争收缴率达95%；及时做好工伤人员认定和伤残鉴定工作； 完善城镇居民医疗保险工作机制，实施城镇居民基本医疗保险市级统筹工作，开展城镇职工医疗保险市级统筹工作；积极开展农村基本养老保险试点工作。努力使广大群众“老有所养、病有所医”。

三是完善社会救助体系。 提高城乡低保保障标准，并相应提高城乡低保对象人均补助水平；将农村五保户供养标准每人每年提高60元。进一步提高精简退职老弱残职工补助、城镇大集体困难企业未参保退休职工补助、国有农林水困难企事业单位未参保退休职工补助、有关企业军转干部和城镇退役士兵自谋职业补助标准、重点优抚对象抚恤补助标准、义务兵家庭优待金标准，适当提高农村“两老 ” 人员补助标准，让每一位困难群众都能分享到改革发展的成果，都能感受到党和政府的关怀和温暖。

四是提高医疗保障水平。 继续实施“光明·微笑”工程，为具备手术适应症的白内障和唇腭裂患者免费治疗。将城镇居民基本医疗保险补助提高到120元，医疗费报销比例一、二、三级医疗机构分别提高至80％、70％和60％；全额资助在校大学生参加属地的城镇居民基本医疗保险；新型农村合作医疗筹资标准由年人均100元提高到150元，住院补偿封顶线由3万元提高到5万元，统筹基金使用率达到85％以上，对农村孕产妇实行住院分娩补助；提高城镇职工医疗保险报销比例。扩大城乡医疗救助覆盖面，逐步提高救助水平。将医疗保险范围进一步扩大到困难农林水企事业单位、困难农垦企业和城镇困难大集体企业职工，适当增加保险补助，切实解决人民群众看病难的问题和因病致贫、因病返贫的问题，不断提高人民群众的健康水平。

五是提高教育保障和公共文化福利水平。 继续完善“奖、贷、补、助、免相结合，大、中、小学校全覆盖”的济困助学体系，为中等职业教育学校涉农专业学生免除学杂费；重点支持地质灾害易发地区校舍改造；继续支持“农家书屋”、自然村通广播电视、乡镇综合文化站及群众体育综合健身场所建设，继续免费为农民群众送演出、送电影，开展文明健康的文化活动，不断满足群众日益增长的精神文化生活需求。

六是加大保障性住房建设力度。 全市新建廉租住房3526套，租赁补贴12967户，改造棚户区危旧房面积76.8万平方米。其中中心城区新建廉租住房1300套，努力让广大低收入家庭住房条件得到明显改善。

七是着力推进扶贫开发。 抓好610个扶贫开发工作重点村整村推进扫尾和验收，完成6000名扶贫移民搬迁任务，加快实施50万亩优质泡桐项目，扎实推进社会扶贫和21万库区移民动态管理和后期扶贫工作。

八是继续改善农村生产生活条件。 继续抓好农村公路和渡改桥建设，全年完成新建水泥（油）路面1100公里，新增通水泥（油）路行政村157个，加快实施53座渡改桥项目。 抓紧实施农村人口饮水安全工程。让农民群众都能走上平坦路、喝上清洁水。

（九）以稳定和谐为目标，力促社会建设更全面、更协调

加快发展社会各项事业，真正让广大百姓公平地享受社会公共服务和产品，在发展中获得实惠，在稳定中共享和谐。

优先发展教育事业。 进一步落实教育优先发展战略， 不断深化教育改革，加快调整城乡学校布局，促进教育均衡发展，加快实施中小学校舍安全工程，改善城乡教育办学条件。继续扶持上饶职院做大做强，继续支持上饶师院加快建设，继续抓好上饶医专设置申报工作 。

加快发展文化体育事业。 深入推进“高雅艺术进上饶” 活动，不断丰富群众文化生活，着力提升城市文化品位。 积极推进广播影视、新闻出版等领域产品创新， 提高公共文化服务水平。举办第二届农民艺术节。 加快乡镇综合文化站、基层文化阵地建设，深入开展农村文化“三项活动”， 完成第三次文物普查。 积极 参加江西省第十三届运动会，贯彻落实《全民健身条例》，推动全民健身运动 。

继 续加强卫生服务能力建设。 大力推进公共卫生均等化，实施好5项 重大公共卫生服务项目和9项基本公共卫生服务项目。继续 做好各种传染病防控工作。做好血防工作，确保信州区、上饶县、广丰县达到传播阻断标准。 加大食品药品监管执法力度，切实保障食品药品安全。

统筹解决人口问题。 坚决兑现计划生育“一票否决”制度，认真落实好各项计划生育奖励扶助政策， 加强出生人口性别比综合治理。 加强生殖健康服务，大力实施“优生工程”，切实 提高人口素质。

全力维护社会安全稳定。 落实安全生产责任制，加强重点行业、重点领域的安全监管，坚决杜绝重特大事故发生。积极开展普法教育、法律服务、法律援助和社区矫正工作，加强基层民主政治建设，营造良好的民主法制环境。围绕深入推进社会矛盾化解、社会管理创新、公正廉洁执法三项重点工作，大力开展社会矛盾纠纷排查化解工作，畅通群众利益诉求渠道，及时解决群众合理诉求，坚决防止发生重特大群体性事件。深化平安创建活动，加强社会治安综合治理，严厉打击各类违法犯罪行为，增强人民群众安全感。强化应急宣教演练，妥善处置各类突发事件，进一步提高应急管理水平和能力。继续深入开展全民国防教育，认真做好人民武装和民兵预备役工作，积极开展创建全国双拥模范城工作，进一步巩固军政军民团结。

（十）以转变作风为抓手，力促政府自身建设更务实、更高效

战胜各种困难和挑战，实现发展目标任务，政府肩负着重要责任。各级政府要努力建设顺应时代要求、适应科学发展、依法阳光行政、符合人民意愿的人民满意政府。

更加注重依法行政。 自觉接受人大及其常委会法律监督和政协民主监督，广泛听取各民主党派、工商联和无党派人士的意见和建议，认真办理人大代表提出的议案、建议和政协提案，加强与工会、共青团、妇联等群团组织的联系，不断改进政府工作。进一步加强行政监察和审计监督，重视和支持人民群众和新闻舆论监督。加强政府法制工作，依法开展行政复议工作，全面推进依法行政，保障公民、法人和其它组织的合法权益，不断提高政府的公信力。

更加注重高效施政。 以开展“创业服务年”活动为契机，扎实推进行政审批职能向一个科室集中、办理服务向一个窗口集中，积极推行网上审批和电子监察。严格执行首问负责制、限时办结制、责任追究制“三项制度”，切实提高行政效能。加大调查研究和监督检查力度，推动工作落实，保证政令畅通。坚持高标准、严要求，保持高效率、快节奏，做到工作程序简化、工作部署简明、召开会议简短、文字材料简练、深入基层简从，把更多的时间和精力用于解决问题、推动发展。

更加注重为民执政。 群众是真正的英雄。我们要虚心向群众学习，善于从群众中汲取经验、获取智慧。凡涉及发展与改革的重大决定，我们都要充分顺应群众的意志；凡涉及社会大局的重要部署，我们都要充分听取群众的意见；凡涉及群众切身利益的重要事项，我们都要充分尊重群众的意愿。各级政府及其工作人员要带着深厚感情，到群众中去，对群众反映的问题，要勇于担当，绝不回避，真心诚意帮助解决，努力践行执政为民宗旨。

更加注重廉洁从政。 扎实推进惩治和预防腐败体系建设，深入开展反腐败斗争，认真落实领导干部、公务员廉洁从政的各项规定，切实提高拒腐防变能力，做到既要干事又要干净。切实抓好资源出让、工程招投标、产权交易等公共资源管理和权力运行的监管，严肃查处各类违纪违法案件，坚决纠正损害群众利益的不正之风。坚持勤俭办事业，珍惜民资民力，精打细算，严禁铺张浪费，切实把有限的资金用在提高群众生活和增强经济发展后劲上。

各位代表，人民的期盼，是我们攻坚克难、奋勇前行的强大动力；人民的信任，是我们加快崛起、成就事业的坚强后盾。让我们更加紧密地团结在以胡锦涛同志为总书记的党中央周围，在中共上饶市委的坚强领导下，深入贯彻落实科学发展观，求真务实，开拓创新，为开创上饶更加美好的未来而努力奋斗！
